# Citrate-Coated Superparamagnetic Iron Oxide Nanoparticles Enable a Stable Non-Spilling Loading of T Cells and Their Magnetic Accumulation

**DOI:** 10.3390/cancers13164143

**Published:** 2021-08-17

**Authors:** Philipp Boosz, Felix Pfister, Rene Stein, Bernhard Friedrich, Lars Fester, Julia Band, Marina Mühlberger, Eveline Schreiber, Stefan Lyer, Diana Dudziak, Christoph Alexiou, Christina Janko

**Affiliations:** 1 Department of Otorhinolaryngology, Head and Neck Surgery, Section of Experimental Oncology and Nanomedicine (SEON), Else Kröner-Fresenius-Stiftung Professorship, Universitätsklinikum Erlangen, 91054 Erlangen, Germany; philipp.boosz@fau.de (P.B.); felix.pfister@uk-erlangen.de (F.P.); rene.stein@uk-erlangen.de (R.S.); bernhard.friedrich@uk-erlangen.de (B.F.); julia.band@uk-erlangen.de (J.B.); marina.muehlberger@uk-erlangen.de (M.M.); eveline.schreiber@uk-erlangen.de (E.S.); stefan.lyer@uk-erlangen.de (S.L.); christoph.alexiou@uk-erlangen.de (C.A.); 2 Friedrich-Alexander-Universität Erlangen-Nürnberg (FAU), 91054 Erlangen, Germany; 3 Institute of Anatomy and Cell Biology, Friedrich-Alexander-Universität Erlangen-Nürnberg (FAU), 91054 Erlangen, Germany; lars.fester@uk-erlangen.de; 4 Laboratory of Dendritic Cell Biology, Department of Dermatology, Universitätsklinikum Erlangen, 91054 Erlangen, Germany; diana.dudziak@uk-erlangen.de; 5 Deutsches Zentrum Immuntherapie (DZI), 91054 Erlangen, Germany; 6 Comprehensive Cancer Center Erlangen-EMN (CCC ER-EMN), 91054 Erlangen, Germany; 7 Medical Immunology Campus Erlangen, 91054 Erlangen, Germany

**Keywords:** nanomedicine, superparamagnetic iron oxide nanoparticles (SPIONs), adoptive T cell transfer, immune therapy, targeted transport, solid tumor, magnetic targeting

## Abstract

**Simple Summary:**

In cancer patients, adoptive T cell transfer shall increase the number of circulating cytotoxic T cells to foster anti-tumor immune responses. In solid tumors, however, lack of lymphocyte infiltration into the tumor impairs treatment efficacy due to the immune-suppressive tumor microenvironment. To make cells controllable by external forces, we loaded primary human T cells with citrate-coated superparamagnetic iron oxide nanoparticles (SPIONs). SPIONs were tightly attached to the plasma membrane and also taken up intracellularly into vesicles. With their nanoparticle cargo, we were able to magnetically accumulate them, which is a promising finding for future magnetic delivery of immune cells after adoptive transfer.

**Abstract:**

T cell infiltration into a tumor is associated with a good clinical prognosis of the patient and adoptive T cell therapy can increase anti-tumor immune responses. However, immune cells are often excluded from tumor infiltration and can lack activation due to the immune-suppressive tumor microenvironment. To make T cells controllable by external forces, we loaded primary human CD3+ T cells with citrate-coated superparamagnetic iron oxide nanoparticles (SPIONs). Since the efficacy of magnetic targeting depends on the amount of SPION loading, we investigated how experimental conditions influence nanoparticle uptake and viability of cells. We found that loading in the presence of serum improved both the colloidal stability of SPIONs and viability of T cells, whereas stimulation with CD3/CD28/CD2 and IL-2 did not influence nanoparticle uptake. Furthermore, SPION loading did not impair cytokine secretion after polyclonal stimulation. We finally achieved 1.4 pg iron loading per cell, which was both located intracellularly in vesicles and bound to the plasma membrane. Importantly, nanoparticles did not spill over to non-loaded cells. Since SPION-loading enabled efficient magnetic accumulation of T cells in vitro under dynamic conditions, we conclude that this might be a good starting point for the investigation of in vivo delivery of immune cells.

## 1. Introduction

Cancer is the second leading cause of mortality worldwide with over 8.7 million deaths in 2016 and is expected to become the leading one by 2060 [1]. Despite death rates have been steadily declining in the last years, treatment is still complex. Surgery, chemotherapy, radiotherapy, alone or in combination, have represented the main treatment options for a long time. Since the immune system itself can recognize and eliminate tumor cells, immune therapy has evolved. Infiltration of solid tumors with immune cells has turned out to be an important criterion for therapy as well as for patient prognosis [2,3]. Especially CD8+ T cells among the tumor-infiltrating lymphocytes (TILs) have a favorable impact on disease burden and progression [4,5]. Adoptive transfer of immune effector cells shall increase the amount of tumor-infiltrating lymphocytes to boost the anti-tumor immune response. This procedure utilizes the isolation of peripheral T cells or TILs from a patient and the expansion and modification to increase the immunogenic anti-tumor activity [6]. However, it has been shown that after the transfer of the T cells only a small number infiltrated into the tumor, resulting in only minor anti-tumor effects [7,8]. Irregular tumor vasculature, barrier function of the tumor epithelium, low expression of adhesion molecules, as well as low chemokine expression in the tumor and/or aberrant chemokine receptor expression on the T cells are discussed to hinder the infiltration of effector cells [9]. To overcome these issues, several very specific strategies dependent on the tumor and T cell type have been developed such as the use of antibodies or homing peptides, which increased activation of the cells but also induced severe adverse events such as cytokine release syndrome or seizures and cerebellar effects [10,11,12]. In addition, their specificity, short half-time, and high costs may represent an obstacle for wider use [13]. Thus, a safe and targeted approach, applicable to various effector cell types and tumors is of urgent need.

Superparamagnetic iron oxide nanoparticles (SPIONs) have come into focus as magnetically controllable shuttles to deliver drugs specifically to the desired region and sparing healthy tissues, referred to as “Magnetic Drug Targeting (MDT)” [14]. For this, SPIONs loaded with a (chemo-)therapeutic drug, are applied to the tumor supplying vascular system and enriched in the tumor region by application of an external magnetic field. We and others have shown successful targeting and enhanced anti-tumor activity of SPION-loaded chemotherapeutic agents when magnetically accumulated in the tumor region [14,15,16]. Following the principle of magnetic targeting, it is not only possible to transport active ingredients to a target by magnetic control. Cells can also be loaded with SPIONs and moved to the desired region by an external magnetic field. For that, the SPIONs must either bind to the cell surface or be taken up into the cell. Dendritic cells, stem cells, and endothelial cells have been functionalized with SPIONs and were magnetically guided to the target tissue for tumor vaccination, to control tissue injury or application in regenerative medicine [17,18,19].

Concerning diagnosis, magnetic nanoparticles can be visualized in magnetic resonance imaging (MRI) and have been used for liver imaging previously. Analogously, SPION-loaded cells can be visualized by MRI, which has been used to track the path of T cells for a better understanding of migration and survival of antigen-specific T cells under pathophysiological situations [20,21,22]. Interestingly, for future translation into clinics, the magnetic field coils inherent to clinical MRI scanners can not only be used for tracking but also steering magnetic nanoparticles or nanoparticle-loaded cells into the wanted region [23,24].

To enable their site-directed targeting, lymphocytes such as NK cells and T cells have been previously functionalized with SPIONs [25,26,27,28,29,30,31,32]. Physicochemical factors of the nanoparticles such as surface charge, size, shape, and coating were reported to influence nanoparticle uptake and biocompatibility [33]. Since lymphocytes are not phagocytic, their nanoparticle uptake is usually low [34], which might reduce magnetic retention. Positively charged coatings of the SPIONs such as 3-aminopropyl-triethoxysilane can enhance interaction with the negatively charged plasma membrane [31] but might also lead to nanoparticle clusters that can detach and spill from the loaded cells. Moreover, NK cells were loaded with positively charged magnetic nanocomplexes and enabled image-based guiding by MRI after intra-arterial infusion [35]. Another employed strategy was the binding of magnetic nanoclusters onto T cells via a PD-1 antibody, which enabled efficient recruiting of T cells to tumor sites, where the PD-1 antibody was released and both, together, exerted a synergistic anti-tumor effect [28]. Zupke et al. showed that the loading efficacy of T cells with nanoparticles strongly depended on nanoparticle concentration, incubation time, and temperature, as well as the presence of serum in the incubation media [36]. The question if SPIONs spill from labeled cells to other cells and the stability of SPIONs in the loaded cells, however, has not been intensively investigated so far.

Besides other challenges, for magnetic guidability the basis is the stable and sufficient loading of SPIONs on or into the T cells without compromising their viability and effector functions. Here, we investigated, with negatively charged citrate-coated SPI-ONs, how experimental conditions influence nanoparticle uptake and viability of primary human T cells.

## 2. Materials and Methods

### 2.1. Materials

Cell culture plates were purchased from TPP (Trasadingen, Switzerland). Merck (Darmstadt, Germany) provided the Muse^®^ Count & Viability Assay Kit. Ringer’s solution was acquired from Fresenius Kabi (Bad Homburg, Germany). Hoechst 33342 (Hoe), 1,1′dimethyl-3,3,3’,3’-tetramethylindodicarbocyanine iodide (DiIC1(5)), Gibco™ RPMI medium 1640, GlutaMAX supplement, penicillin-streptomycin solution 5000 U/mL, Vybrandt™ DiD, Syto16, Fixable Viability Dye eFluor™ 450, CellTrace™ Violet (CTV), and L-glutamine (200 mM) were purchased from Thermo Fisher Scientific (Waltham, MA, USA). Propidium iodide (PI) and phosphate-buffered saline (PBS) were obtained from Sigma-Aldrich (Taufkirchen, Germany), fetal calf serum (FCS), and amphotericin B from Biochrom (Berlin, Germany).

The Human CD3 Fab-TACS Gravity Kit was obtained from IBA (Goettingen, Germany), and the nitric acid 65 % from Carl Roth (Karlsruhe, Germany). The H_2_O used in all experiments was prepared in-house using the Merck Milli-Q^®^ Direct water purification system (Darmstadt, Germany). Falcon^®^ 40 µm and 70 µm cell strainers were purchased from Corning by Life Sciences (Corning, NY, USA) and LabSolute (Renningen, Ger-many). Annexin A5 fluorescein isothiocyanate (FITC) conjugate (AxV-FITC) and recombinant human Interleukin (rhIL)-2 and rhIL-7 were obtained from ImmunoTools (Friesoythe, Germany), the ImmunoCult™ Human CD3/CD28/CD2 T cell Activator was obtained from Stemcell Technologies (Vancouver, BC, Canada), and the S-Monovettes 10 mL 9 NC were obtained from Sarstedt (Nuembrecht, Germany). BD Insyte-W intravenous cannula, 16GA were purchased from BD (Haryana, India). Iron reference standards (1 g/L) were purchased from Bernd Kraft GmbH (Duisburg, Germany).

BioLegend (San Diego, CA, USA) provided APC anti-human CD8a, APC Mouse IgG1, κ Isotype Control, PerCP/Cy5.5 anti-human CD4, PerCP Mouse IgG1, κ Isotype Control, and PE anti-human IL-2. FITC mouse anti-human CD3, Pacific Blue mouse anti-human CD8, Pacific Blue mouse IgG1, κ, Isotype Control and FITC Mouse IgG1, κ, Isotype Control were obtained from BD Pharmingen (San Jose, CA, USA). Inside Stain Kit, PE anti-human Tumor Necrosis Factor (TNF)-α, APC-Vio 770^®^ anti-human Interferon (IFN)-γ were purchased from Miltenyi Biotec (Bergisch Gladbach, Germany). Ibidi (Gräflingen, Germany) provided µ-Slide I Luer with a channel height of 0.4 mm. The peristaltic pump Ismatec^®^ IPC and PharMed^®^ BPT tubes (2.06 mm inner diameter) were obtained from Cole-Parmer GmbH (Wertheim, Germany). Neodymium disc-shaped magnets (5 mm × 5 mm, approximately 400 mT) were purchased from Webcraft GmbH (Gottmadingen, Germany).

The Gallios flow cytometer and Kaluza analysis Software (1.3, 2.1) were obtained from Beckman Coulter (Brea, CA, USA). The SpectraMax iD3 plate reader was purchased from Molecular Devices (San José, CA, USA).

For the analysis of T cells in transmission electron microscopy (TEM) the following was used: Carl Roth (Karlsruhe, Germany) supplied glutardialdehyde 25 % (for microscopy) in PO_4_-buffer, ethanol (absolute), acetone (100 % anhydrous), hardener MNA, DPA, glycidyl ether (100 %), accelerator DMP 30, and NaOH (1 M). Science Service (Munich, Germany) supplied OsO_4_, copper grid. Merck (Darmstadt, Germany) supplied K_3_(Fe(Cn)_6_, agarose (low melting point), and lead(II)citrate-3-hydrate. Serva (Heidelberg, Germany) supplied uranyl acetate. The following equipment from these companies was used: Emerson (Saint Louis Missouri, United States) Branson 1200 ultra-sonic machine, Leica (Wetzlar, Germany) Ultracut UCT, Zeiss (Oberkochen, Germany) TEM Leo 906, TRS Tröndle (Moorenweis, Germany) CCD camera, ImageSP SysPROG.

### 2.2. Synthesis of SPIONs and Physicochemical Characterization

SPIONs were synthesized based on an adjusted protocol of Elbialy et al. [37] in three batches. Particles were sterilized by filtration through syringe filters with 0.2 µm pore size (Sartorius, Goettingen, Germany). Subsequently, SPIONs were analyzed regarding their size, iron content, magnetic susceptibility, and zeta potential according to Mühlberger et al. [26]. The iron content was investigated after a dilution of 1:25 in deionized H_2_O and dissolving in 65 % nitric acid with atomic emission spectroscopy (AES), using the Agilent 4200 MP-AES (Agilent Technologies, Santa Clara, CA, USA) with an iron solution of 1000 mg/l as an external standard (Bernd Kraft, Duisburg, Germany). Triplicate measurements were performed at a wavelength of 371,993 nm, which were then averaged.

### 2.3. Isolation of T Cells from Human Whole Blood

Human cells were isolated from peripheral human blood obtained from healthy volunteers after informed consent (approved by the ethics committee of the Friedrich-Alexander-Universität Erlangen-Nürnberg; reference number 257_14 B). To obtain CD3+ T cells from citrate-anticoagulated human blood, the CD3 Fab-TACS™ Gravity Kit was used according to the manufacturer’s instructions for freshly drawn blood. Isolated T cells were counted in a MUSE cell Analyzer using the MUSE^®^ Count and Viability assay kit.

### 2.4. Determination of T Cell Purity

To compare the T cell frequency in whole blood before isolation with the one after isolation, erythrocytes were lysed in 100 µL whole blood for 20 s using 600 µL 0.12 % formic acid (pH 2.7). Immediately after lysis, the solution was neutralized with 265 µL solution containing 6 g/L sodium carbonate, 14.5 g/L sodium chloride, and 31.1 g/L sodium sulphate (pH 11.2). Then, the cells were washed with 1 mL PBS and centrifuged at 300 rcf for 5 min at room temperature. Lysed whole blood and freshly isolated T cells were then stained with antibodies against CD3, CD4, CD8, and corresponding isotype controls for 30 min at 4 °C. Subsequently, the cells were investigated by flow cytometry and the data was analyzed with the Kaluza software (version 2.1).

### 2.5. Determination of Cell Viability

Cell viability was determined in flow cytometry by staining 50 µL cell suspension with 250 µL of staining mixture for 20 min at 4 °C. The staining mixture contained 20 nM Hoe, 2 µL/mL AxV-FITC, 4 nM DiIC1(5), and 66.6 ng/mL PI per ml Ringer’s solution. Cells were analyzed in a Gallios flow cytometer. Electric compensation was used to eliminate fluorescence bleed through. Data were analyzed with the Kaluza software (version 2.1).

### 2.6. Colloidal Stability of SPIONs in Various Media and Cellular Nanoparticle Uptake

If not indicated otherwise, T cells were cultured in a humidified 5% CO_2_ atmosphere at 37 °C in a standard T cell medium composed of RPMI 1640 medium supplemented with 10% heat-inactivated (HI) FCS, 2% penicillin-streptomycin solution, 1% L-glutamine, and 1% amphotericin B.

To investigate the colloidal stability dependent on the medium composition, nanoparticles (0, 26.7, 53.3, 80.0, and 106.7 µg/mL) were incubated in 1) T cell medium with 10% FCS or 2) T cell medium with only 2% FCS or 3) PBS with 2% FCS overnight in cell culture conditions. The next day, 100 µL supernatant of each sample, as well as 100 µL of remaining fluid with resuspended SPIONs, was transferred to separate wells of a 96-well plate. The optical density of the samples was measured at 320 nm in the SpectraMax iD3. To analyze the formation of SPION agglomerates depending on the medium composition, 50 µL aliquots of each well were added to 250 µL of Ringer’s solution and then analyzed by flow cytometry (forward scatter: voltage 500, gain 5, discriminator 20; side scatter: voltage 550, gain 10, discriminator off).

To evaluate the effect of the medium composition on the uptake of SPIONs by T cells, 1 × 10^6^ freshly isolated T cells in 100 µL were incubated with SPIONs for one hour. Then, cells were stained for viability as described above, analyzed by flow cytometry, and their iron content was quantified in AES as detailed in Section 2.2.

### 2.7. Comparison of Stimulated and Non-Stimulated T Cells

2 × 10^6^ of T cells were stimulated in a concentration of 1 × 10^6^ cells per ml with 30 IU/mL rhIL-2 and 25 µL/mL/1 × 10^6^ cells of Immunocult™ human CD3/CD28/CD2 T cell activator. Unstimulated CD3-positive cells in standard T cell medium served as controls. After 72 h, 2 mL of either the T cell medium only or the T cell medium containing 30 IU/mL rhIL-2 was added to the controls or stimulated T cells, respectively.

To determine the effects of the stimulation on the viability of the T cells, flow cytometry was performed after 0 h and 96 h as described above. Additionally, MUSE cell count and viability analyses were conducted to confirm the flow cytometry results.

### 2.8. Determination of Nanoparticle Uptake

T cells were stimulated as described in Section 2.7. After the second stimulation at 72 h, 200 µL SPION solution was added to the test samples or the controls, respectively. A control was also established, which received only 200 µL deionized H_2_O without SPIONs. After an additional 24 h, the T cells were washed twice with PBS to remove free nanoparticles. The T cell number was determined via the Muse Cell Analyzer. Cells were then sedimented by centrifugation at 300 rcf for 5 min. After discarding the supernatant, the pellets were dried at 95 °C, 300 rpm for 30 min in a 1.5 mL Eppendorf tube, before they were lysed with 100 µL nitric acid at 95 °C, 750 rpm for 15 min. 900 µL of water was added, followed by measurement of their iron concentration by AES.

The long-term stability of the ingested SPIONs was also investigated. T cells were loaded with 80 µg Fe/mL SPIONs overnight, controls received only deionized H_2_O. The cells were then seeded at a concentration of 2 × 10^6^ cells in 2 mL of T cell medium. 10 ng/mL IL-7 was added to the T cell medium to increase long-term survival. After 24 h, 48 h, and 72 h, the cells were collected, washed three times to remove any unbound iron, and cell count was determined using the MUSE Cell Analyzer. Samples were dried at 95 °C for 30 min, lysed with 50 µL HNO_3_ at 95 °C for 15 min, and dissolved in 450 µL deionized H_2_O. The iron content was determined with AES and the pg iron per cell was calculated by dividing the iron amount by the cell number.

### 2.9. SPION Exchange with Non-Loaded T Cells

2 mL of 1 × 10^6^ CD3+ T cells per 1 mL in T cell medium were seeded in 12-well plates, half of them were incubated with SPIONs overnight; the control group was treated the same way with deionized H_2_O. The next day, nanoparticles were washed from the samples at 300 rcf for 5 min at room temperature. Loaded T cells were stained with FITC anti-human CD3 antibody for 30 min at 4 °C with a working solution of 1:200 in T cell medium, were washed two times and resuspended. Non-loaded cells were resuspended in medium and placed into 96-well plates. Triplicates of 100 µL pure non-loaded T cells, triplicates of 100 µL loaded T cells, and triplicates with mixed 50 µL non-loaded and 50 µL stained loaded T cells were placed into 96-well plates and incubated for three hours in an incubator at 37 °C. All samples were placed in the flow cytometer and the side scatter was analyzed. Populations of non-loaded and loaded CD3+ T cells were differentiated by a CD3 stain.

To investigate the long-term stability of the ingested iron in the cells, T cells were stained with 5 µM of CTV for 30 min at 37 °C and then loaded with 80 µg Fe/mL SPIONs overnight as described above; an unstained control received only deionized H_2_O. The cells were then washed to remove excess particles and seeded in a 12-well plate at a concentration of 1 × 10^6^ cells in 2 mL of T cell medium. IL-7 was added to the T cell medium at a concentration of 10 ng/mL, to increase the long-term survival. The exchange between loaded and non-loaded cells was investigated by the seeding of 0.5 × 10^6^ of each loaded and non-loaded T cells in 2 mL of T cell medium. After 24 h, 48 h, and 72 h, cells were collected, stained with 2 µL/mL AxV-FITC and 66.6 ng/mL PI for 20 min at 4 °C, and then analyzed by flow cytometry.

### 2.10. Transmission Electronic Microscopy

For determination of the incorporated amount of SPIONs, transmission electronic microscopy (TEM) was performed. For that, T cells were isolated and incubated for 72 h in T cell medium. Afterwards, SPIONs were added to 8 × 10^6^ T cells per sample for 24 h to receive 2667 µg/mL or 80 µg/mL of iron. Cells that received only H_2_O without SPIONs served as controls. Subsequently, the cells were washed three times with PBS whereupon they were fixated in 2.5 % glutaraldehyde in 0.1 M PO_4_ buffer for 4 h, subsequently washed 3 times with 0.1 M PO_4_ buffer and left overnight at 4 °C. The staining of the cells for TEM was performed with 1% osmium treta oxide (OsO_4_) in 3% potassium ferricyanide (K_3_(Fe(Cn)_6_) for 2 h at room temperature (RT), and additionally washing the stained cells with 0.1 M PO_4_ buffer overnight at 4 °C. Before embedding the cells in Epon resin, the cells were transferred into a matrix of 2% agarose (low melting point) in 0.1 M PO_4_ buffer in an Eppendorf reaction tube overnight. For Epon resin embedding, the cells were dehydrated in agarose through an ethanol step and finally transferred to Epon using acetone and an acetone–epon mixture, followed by a polymerization step for 48 h at 60 °C. Ultra-thin sections of approximately 50 nm section thickness of the samples were prepared with the Leica Ultracut UCT and placed on copper grids. These ultra-thin sections were then first contrasted with lead citrate for 10 min and then with uranyl acetate for a further 10 min. The images were taken with the Zeiss TEM 906 LEO (from Zeiss) with a CCD-camera residual light amplifier from A. Tröndle, TRS, and the software ImageSP SYS Prog, TRS. The images were taken at an acceleration voltage of 80 kV and a magnification of 12,930-fold.

### 2.11. Magnetic Accumulation of SPION-Loaded T Cells under Dynamic Conditions

The magnetic attractability of CD3+ T cells after SPION-loading was evaluated under dynamic conditions. To imitate blood flow, a peristaltic pump was used to move SPION-loaded cells through slides to which Neodymium disc shape magnets (5 mm × 5 mm, approximately 400 mT) were attached. Unloaded cells and slides without magnets served as controls. After 1 h of pumping, magnetically accumulated cells were stained with the Hoechst 33,342 stain. Images were taken on a Zeiss Axio Observer Z1 fluorescence microscope (Carl Zeiss AG, Oberkochen, Germany). Subsequently, pictures were analyzed using the ImageJ software (version 1.52a).

### 2.12. T Cell Activation and Proliferation after Polyclonal Stimulation

The T cells were investigated for cytokine production after stimulation. T cells were isolated and loaded as described above and stimulated with 25 µL per 1 × 10^6^ cells per 1 mL ImmunoCult human CD3/CD28/CD2 T-cell activator mix, however, no IL-2 was added. After 24 h, the T cells were stained for CD4 and CD8 for 30 min at 4 °C, in order to distinguish T cell subclasses. Afterwards, the cells were fixated and permeabilized with the inside stain kit. The fixation was performed at room temperature for 20 min with 100 µL of Inside Fix. Cells were then permeabilized for 30 min at 4 °C with the addition of antibodies against TNF-α, IF-γ, and IL-2. Subsequently, the cells were analyzed by flow cytometry.

### 2.13. Data Analysis and Statistics

Data were prcessed in Microsoft Excel (Microsoft, Redmond, WA, USA). Statistical analysis and graph creation were performed with GraphPad PRISM 9.0.2 from GraphPad Software, Inc. (San Diego, CA, USA). For statistical significance, *p*-values ≤ 0.05 were considered.

## 3. Results

### 3.1. Physicochemical Characterization of SPIONs

Magnetic susceptibility, hydrodynamic Z-average size, and zeta potential of three independently synthesized batches of SPIONs were investigated (Table 1). The magnetic susceptibility of the particles normalized to an iron concentration of 1 mg/mL for the purpose of comparison was determined to be ranging from 4.08 × 10^−3^ to 4.12 × 10^−3^. The hydrodynamic Z-average size of the nanoparticles was in mean 52 nm to 58 nm. In accordance, the polydispersity index (PDI) in water was 0.143 to 0.152. The zeta potential ranged from −48.5 mV to −53.7 mV in deionized H_2_O with a pH of 7.3.

### 3.2. Colloidal Stability of SPIONs in Cell Culture Medium

**Figure 1:**
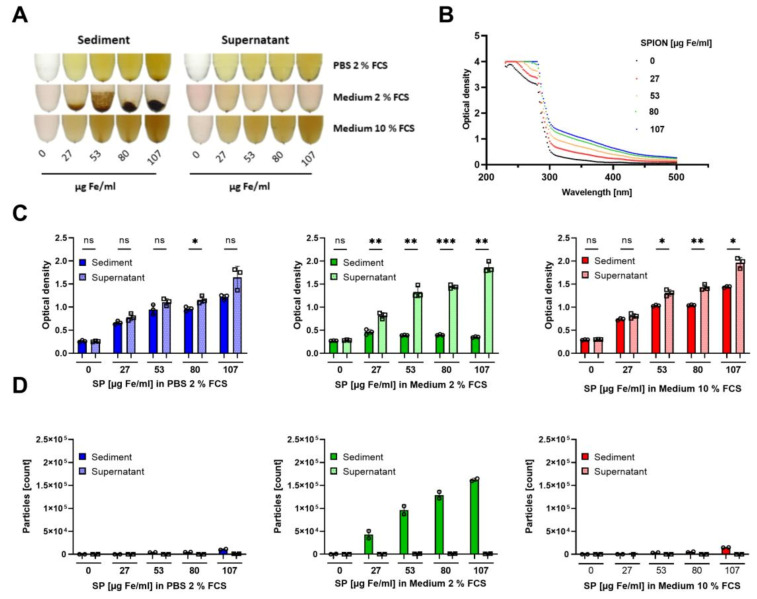
Colloidal stability of SPIONs in various media. SPIONs were incubated overnight in PBS with 2% FCS or RPMI cell culture medium containing 2% or 10% FCS, respectively. (**A**) Macroscopic pictures of SPIONs diluted in PBS supplemented with 2% FCS or T cell medium with 2% or 10% FCS, respectively. (**B**) Spectrum of SPIONs diluted in RPMI with 10% FCS. (**C**,**D**) T cell medium with 10% FCS is depicted in red, while T cell medium with 2% FCS is shown in green. PBS supplemented with 2% FCS is depicted in blue. (**C**) Optical density at 320 nm of the supernatant of sedimented or resuspended samples. (**D**) Supernatant or sediment was analyzed in flow cytometry for agglomerations. Shown are the mean values of triplicates (**C**) or duplicates (**D**) with standard deviations. Significances were calculated using unpaired t-test with Welch’s correction; * *p* ≤ 0.05; ** *p* ≤ 0.01; *** *p* ≤ 0.001. ns: not significant, SP: SPIONs.

Since the protein corona is known to play a crucial role in nanoparticle uptake, we reduced the FCS amount from 10% to 2% in the cell culture medium in order to increase cellular nanoparticles loading. Experiments were additionally performed in 2% FCS in PBS. To determine the colloidal stability of SPIONs in the above-mentioned media, the nanoparticles were incubated overnight to allow them to agglomerate and sediment. On the next day, the nanoparticle suspensions were analyzed for agglomerations. In the medium with only 2% FCS, already macroscopically nanoparticle agglomerations were visible in a dose-dependent manner. In PBS with 2% FCS and medium with 10% FCS, we found no obvious larger nanoparticle sedimentations (Figure 1A). To analyze smaller agglomerations, which were not visible to the naked eye, we measured the absorption of the supernatant of sedimented or resuspended nanoparticle suspensions, respectively. Recording of the spectrum (250–500 nm) of nanoparticle dilutions revealed an optimal wavelength for the detection of SPIONs at 320 nm (Figure 1B). In the case of complete colloidal stability, the absorption of sedimented and resuspended samples should be equal. SPIONs in T cell medium containing 10% FCS and 2% FCS in PBS revealed the best colloidal stability as determined by absorption measurements of the supernatant of the sedimented and resuspended samples. Thus, absorption values of the supernatant of sedimented and resuspended samples increased dose-dependently and were not dramatically different between both. In contrast, the medium containing only 2% FCS induced nanoparticle agglomeration and sedimentation, causing large differences in the optical density values of the supernatants of sedimented and resuspended samples. The resuspended samples induced a dose-dependent absorption, whereas already in the smallest tested concentration the values of the sedimented and resuspended samples were different (Figure 1C). Analyzing the agglomerations by flow cytometry supported this conclusion: only in the T cell medium with 2% FCS nanoparticle agglomerations were detected by their relative size (forward scatter) and granularity (side scatter). In the other solutions, the nanoparticles were colloidally stable and the nanoparticle sizes were below the detection limit (Figure 1D).

### 3.3. Influence of Medium Composition on SPION-Loading of T Cells

T cell isolation via IBA gravity columns resulted in a CD3+ T cell purity of 91.5% ± 1.1% compared to the portion of CD3+ T cells of 34.9% ± 3.9% in whole blood (Figure S1). To achieve magnetic guidability of the cells by loading with SPIONs, a sufficient amount of iron per cell is crucial. Based on previous data by Mühlberger et al. [27], we selected 80 µg/mL as the optimal iron concentration for the loading of T cells. Similar to the loading strategy of Sanz-Ortega [31], we reduced the medium volume, resulting in a cell count of 1 × 10^6^ cells in 100 µL of medium or PBS in the presence of 80 µg/mL SPIONs for 1 h to enhance contact of cells with the nanoparticles.

**Figure 2:**
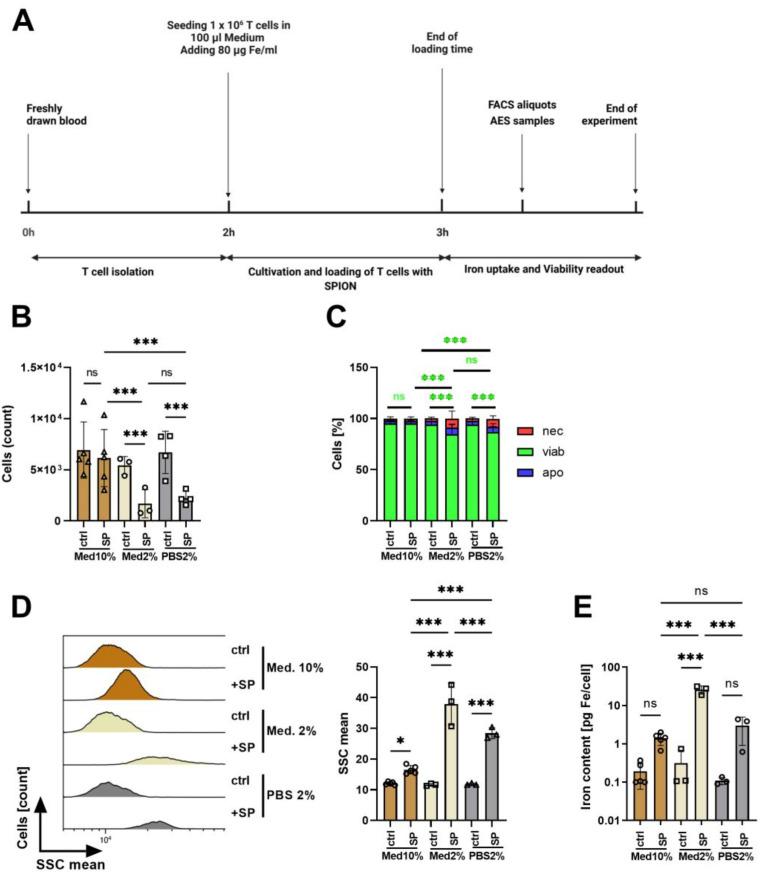
Influence of medium composition on SPION-loading of T cells. (**A**) Experimental setup. (**B**) Determination of the cell count by flow cytometry. (**C**) The viability of the cells was determined by AxV-FITC and PI staining in flow cytometry. AxV-FITC-/PI- cells were considered viable, AxV-FITC+/PI- apoptotic, and PI+ cells necrotic. (**D**) Estimation of cellular nanoparticle loading by analysis of the side scatter increase of viable cells by flow cytometry. (**E**) Cellular iron content [pg Fe/cell] was determined by atomic emission spectroscopy of cell lysates. (**B**–**E**) Experiments were performed in triplicates of T cells from 3–5 donors. Shown are the mean values with standard deviations. Significances were calculated with a 2-way ANOVA test with a Tukey post hoc test; * *p* ≤ 0.05; *** *p* ≤ 0.001. ctrl: control; SSC: side scatter; med: medium; ns: not significant; nec: necrotic; apo: apoptotic; viab: viable.

Subsequently, cells were washed, counted, and the amount of iron was determined from the cell lysates using AES. Cell count and viability were directly (without washing) determined by flow cytometry after staining for apoptosis and necrosis using AxV-FITC and PI (Figure 2A). AxV-FITC stains phosphatidylserine which is exposed on the outer surface of the cells in apoptosis. Counterstaining with the plasma-membrane impermeable dye PI detects necrotic cells. Thus, AxV-FITC-/PI- cells are considered viable, AxV-FITC+/PI- cells apoptotic, and PI+ cells necrotic.

With the medium containing 10% FCS, the cell count was not reduced in the presence of SPIONs. In contrast, SPIONs in both medium or PBS containing 2% FCS reduced the cell count dramatically (Figure 2B), which might be due to iron overload and rupture of cells. The majority of the residual cells, however, were viable (AxV-FITC-/PI-) (Figure 2C).

As shown by Friedrich et al., nanoparticle uptake was accompanied by an increase of side scatter by flow cytometry [38], serving as a marker to estimate SPION-loading of the cells. Since cell death processes also cause the alteration of cell morphology, we gated only on viable cells based on their AxV-FITC and PI negativity. In RPMI medium with 2% FCS, in which the nanoparticles agglomerated, the cells showed the largest side scatter increase. In PBS containing 2% FCS, in which the nanoparticles were colloidally stable, the side scatter increase was smaller, and in medium with 10% FCS hardly detectable (Figure 2D). We analyzed the iron amount in the cell lysates using AES and calculating the iron amount in relation to the cell count. In line with the side scatter data, we achieved the highest iron values per cell for T cells in medium with 2% FCS with 26.03 pg ± 5.32 pg iron per cell, which might be due to nanoparticle agglomerations in the cell lysates. T cell loading in PBS with 2% FCS resulted in 2.96 ± 1.67 pg iron per cell. Cells in the medium with 10% FCS contained only 1.40 ± 0.44 pg iron per cell (Figure 2E).

### 3.4. Influence of Polyclonal Stimulation on SPION-Loading of T Cells

**Figure 3:**
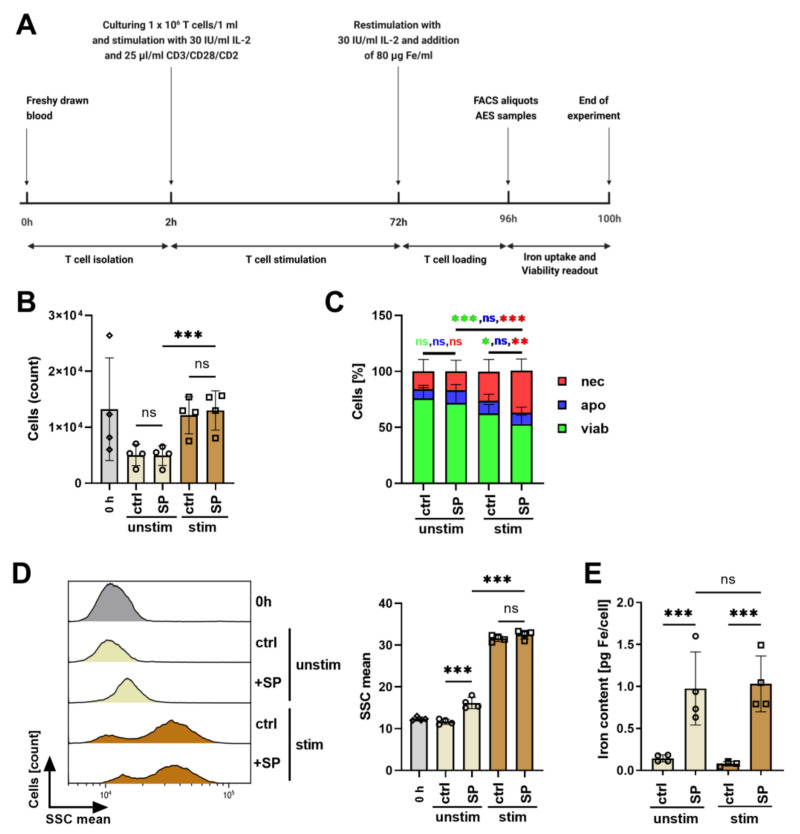
Influence of T cell stimulation on nanoparticle uptake. (**A**) Experimental setup. 1 × 10^6^ CD3+ T cells in 1 mL T cell medium were stimulated for 72 h and then re-stimulated and incubated with 80 µg/mL SPIONs for 24 h. Unstimulated and/or cells without SPIONs served as controls. Subsequently, cells were analyzed by flow cytometry and atomic emission spectroscopy (AES). Determination of cell count (**B**) and viability (**C**) in flow cytometry. AxV-FITC-/PI- cells were considered viable, AxV-FITC+/PI- cells apoptotic, and PI+ cells necrotic. (**D**) Side Scatter (SSC) increase of AxV-FITC-/PI- cells indicates nanoparticle uptake. E) Determination of the iron amount (pg/cell) by AES. (**B**–**E**) Experiments were performed in triplicates of T cells from four donors. Shown are the mean values with standard deviations. Significance was estimated by a 2-way ANOVA with a Tukey post hoc test; * *p* ≤ 0.05; ** *p* ≤ 0.01; *** *p* ≤ 0.001. ctrl: control; ns: not significant; SP: SPIONs, stim: stimulated; unstim: unstimulated; nec: necrotic; apo: apoptotic; viab: viable.

Previously, it has been shown that uptake of nanoparticles by cells is dependent on the cell cycle state, whereas uptake in G2/M was stronger than in the S and G0/G1 phase, respectively [39]. In order to increase nanoparticle uptake by cells, we aimed to foster the division of the cells by polyclonal primary and costimulatory stimulation using CD3/CD28/CD2 beads in the presence of 30 IU/mL rhIL-2 for 72 h (Figure 3A). Then, cells were re-stimulated with 30 IU/mL rhIL-2 for another 24 h. At this time point, 80 µg/mL SPIONs were added as well. Then, after 24 h incubation with SPIONs and an overall 96 h of stimulation, viability and iron uptake were determined by flow cytometry and AES, respectively. At this time point, the stimulated T cells were at the cell division stage. Unstimulated cells served as controls.

Flow cytometry revealed that with stimulation the cell count increased from 5.038 ± 1.637 to 12.154 ± 2.865 cells, whereas the presence of nanoparticles had no influence on the cell count after stimulation (4.932 ± 1.537 to 13.004 ± 3.037 cells) (Figure 3B). The cell viability, however, was reduced by the stimulation from 75.8% ± 9.8% to 62.4% ± 8.0%. The presence of SPIONs further reduced the cell viability of non-stimulated and stimulated cells to 71.8% ± 10.8% and 52.9% ± 8.0%, respectively (Figure 3C).

The side scatter of non-stimulated cells increased from 11.67 ± 0.53 to 16.17 ± 1.17 in the presence of nanoparticles. For stimulated cells, the side scatter of the cells was generally higher, possibly because of alterations in morphology due to the proliferation. Nonetheless, incubation with nanoparticles further significantly increased it from 31.58 ± 0.64 to 32.5 ± 0.89 (Figure 3D). Determination of the iron content in the cell pellets via AES and calculation of the iron content per cell confirmed the nanoparticle loading of the cells in the presence of SPIONs. Interestingly, however, we saw no significant differences in the cellular iron amount dependent on the polyclonal stimulation of the cells. We observed 0.976 ± 0.43 pg Fe/cell for unstimulated and 1.03 ± 0.33 pg Fe/cell for stimulated cells (Figure 3E).

### 3.5. SPION-Loaded T Cells Do Not Exchange Nanoparticles with Non-Loaded T Cells

After nanoparticle loading, it is mandatory that SPIONs are not released from the cells or exchanged with other cells, to minimize loss of magnetic controllability, spill over to formerly non-loaded cells, and the bias of other cells getting also magnetically attracted.

**Figure 4:**
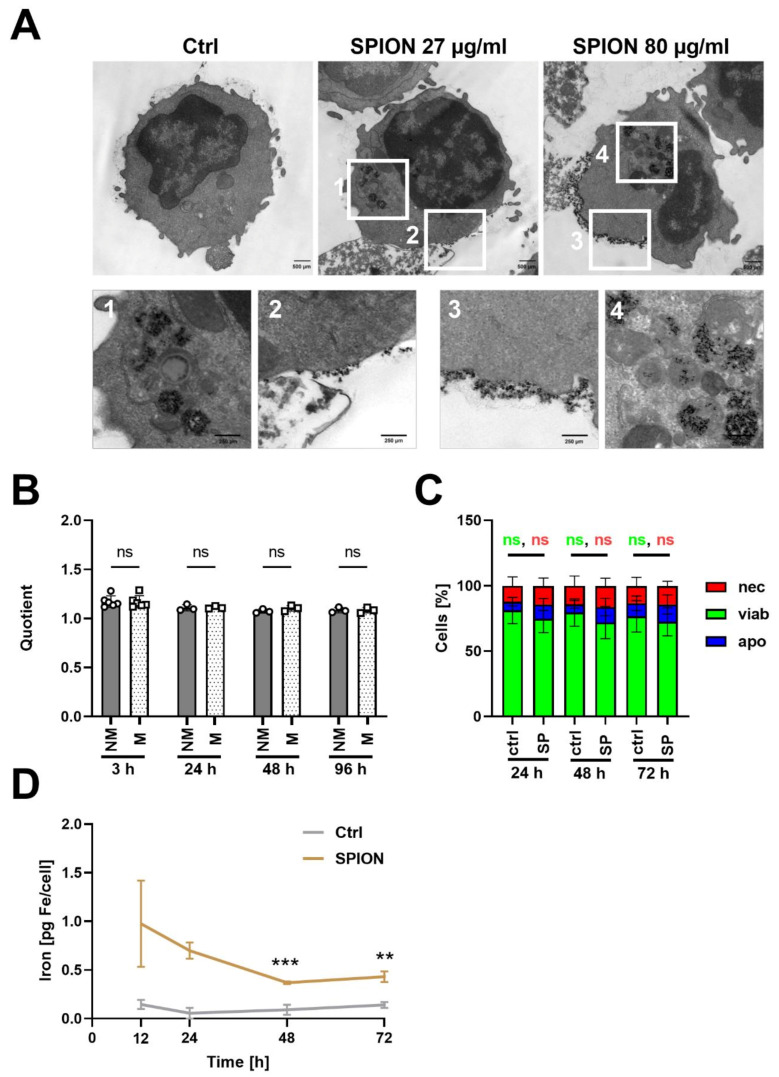
SPIONs were not exchanged between loaded and non-loaded CD3+ T cells. (**A**) Subcellular localization of SPIONs determined by TEM. Scale bars depict 500 µm (upper row) and 250 µm (lower row). Squares indicate areas with SPION-loading in T cells. (**B**) Isolated CD3+ T cells (1 × 10^6^ cells/mL) were loaded with 80 µg Fe/mL SPIONs overnight and stained for CD3 or CTV; non-loaded T cells served as controls. Side scatter values were used for estimation of nanoparticle uptake of SPION-loaded and non-loaded cells. A quotient was calculated by division of the SSC of loaded T cells with that of non-loaded T cells of the same donor at the same time point. (**C**) Cell viability of SPION-loaded cells. Cells were stained with AxV-FITC and PI. Ax-FITC-/PI- cells were considered viable (viab), Ax-FITC+/PI- apoptotic (apo), and PI+ necrotic (nec). (**D**) Iron content per cell as determined by atomic emission spectroscopy and division through cell count. Shown are the mean values with standard deviations. Significances were estimated using an unpaired t test with Welch’s correction; ** *p* ≤ 0.01, *** *p* ≤ 0.001; ctrl: control, SP: SPIONs, NM: not mixed, M: mixed.

Transmission electron microscopy of the unstimulated cells revealed both the binding of the SPIONs to the plasma membrane as well as internalization into vesicles (Figure 4A). With 27 µg/mL of SPIONs, most of the nanoparticles were located intracellularly in vesicles. Only some particles were attached to the plasma membrane. With 80 µg/mL, obviously more nanoparticles were associated with the plasma membrane. These nanoparticles seemed to be tightly attached in a uniform layer at one side of the cell. In the magnification (Figure 4A, picture 3), the formation of a membrane invagination with SPIONs is visible.

To investigate nanoparticle release from SPION-loaded T cells, cells were loaded overnight with 80 µg Fe/mL SPIONs and stained for CD3 or with CTV for identification by flow cytometry. Subsequently, we mixed loaded T cells with non-loaded T cells and analyzed them after 3 h, 24 h, 48 h, and 72 h by flow cytometry regarding SSC increase. Non-loaded T cells and loaded T cells in separate tubes served as controls. For comparison between the time points, a quotient was calculated from the SSC of loaded T cells divided by the SSC of unloaded T cells from the same donor at the same time point. The quotients of non-mixed and mixed samples were not significantly different at each time point (Figure 4B), indicating that nanoparticles were not exchanges between loaded and formerly non-loaded cells. In parallel, we determined the viability after 24 h, 48 h, or 72 h after loading by AxV-FITC and PI staining. As depicted in Figure 4C, after 24 h 81.1% ± 10.1% of unloaded T cells were still viable compared to 74.7% ± 10.5% of loaded T cells. This slightly reduced viability was also detectable after 48 h (non-loaded: 79.5% ± 10.4%, loaded: 71.9% ± 12.4%) and 72 h (non-loaded: 76.7% ± 12.0%, loaded 72.5% ± 10.8%), however was not significant (Figure 4C). Furthermore, the iron content per cell after 24 h, 48 h, and 72 h was determined by AES. Starting with an iron content around 1 pg/cell at 12 h after loading, the iron amount was reduced to 0.5 µg/mL at 48 h until it remained constant (Figure 4D).

### 3.6. Loading of T Cells with SPIONs Allows Magnetic Enrichment under Dynamic Conditions

**Figure 5:**
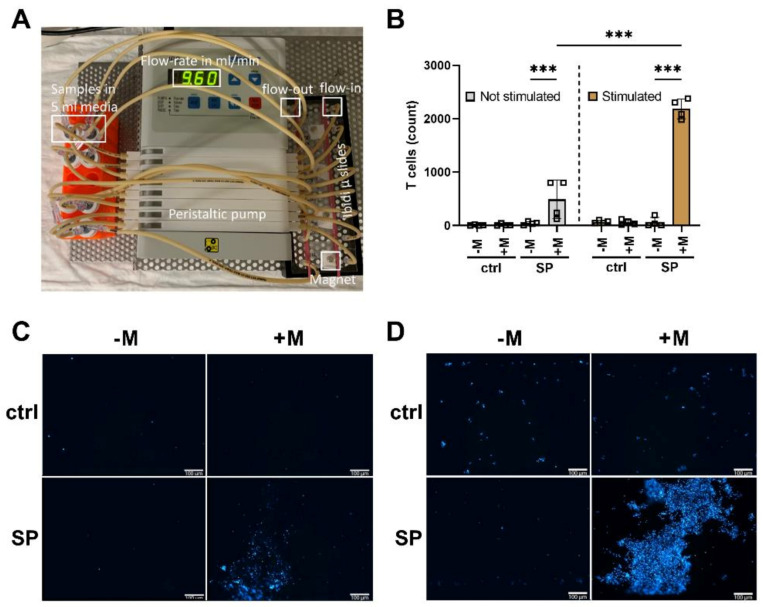
Dynamic magnetic enrichment of T cells. CD3+ T cells were isolated and stimulated for 72 h with a CD3/CD28/CD2 activation mix with 30 IU/mL IL-2. After three days, the T cells received an additional 30 IU/mL IL-2 as well as 80 µg Fe/mL SPIONs for 24 h. T cells without SPIONs or un-stimulated T cells served as controls. Subsequently, the cells were stained with Hoechst 33342. Dynamic enrichment was performed with a peristaltic pump at a flow velocity of 9.6 mL/min for 1 h in µ-slides I Luer. Magnets were added to locally enrich T cells. For the controls, no magnets were added. The slides were then imaged in fluorescent microscopy to analyze cell count. (**A**) Depiction of the experimental setup. (**B**) T cell count after the dynamic enrichment and (**C**,**D**) corresponding fluorescent microscopy images of the area where the magnet was placed of non-stimulated (**C**) and stimulated cells (**D**). Statistical significances were calculated with an unpaired t test with Welch‘s correction; *** *p* ≤ 0.001. ctrl: control; SP: SPION; -M: without Magnet; +M: with Magnet; the white scale bar indicates 100 µm.

The magnetic attractability of CD3+ T cells after SPION-loading was evaluated under dynamic conditions. To imitate blood flow in a physiological vascular system, a peristaltic pump was used to move SPION-loaded cells through slides to which magnets were attached. Non-loaded cells and slides without magnets served as controls (Figure 5A). The catching of SPION-loaded cells (containing roughly 1 pg iron/cell) was visible even macroscopically. After 1 h of pumping, magnetically accumulated cells were analyzed by fluorescence microscopy by Hoechst staining. For cells without SPION-loading, no cell accumulation was detected. With SPION loading, the numbers of cells increased from 62 ± 90 to 2188 ± 186 (stimulation) in the presence of a magnet, proving their magnetic attractability (Figure 5B–D).

### 3.7. SPION Loading Does Not Impair Cytokine Release and Differentiation of T Cells after Polyclonal Stimulation

**Figure 6:**
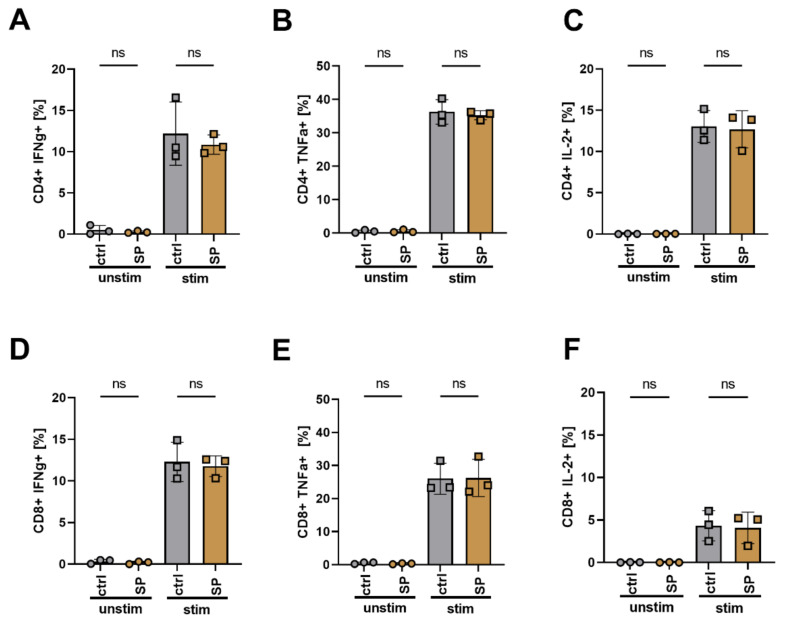
Cytokine expression of T cells after stimulation. Isolated T cells were loaded with 80 µg Fe/mL SPIONs and were stimulated with a CD3/CD28/CD2 activation mix overnight. H_2_O treated cells and cells without stimulation mix served as controls. The cells were then stained for CD4 and CD8, viability, and cytokine expression. Depicted are the percentages of CD4 (**A**–**C**) or CD8 (**D**–**F**) T cells expressing TNFα (**A**,**D**), IFNγ (**B**,**E**) or IL-2 (**C**,**F**). Statistical significances were estimated using an unpaired t test with Welch’s correction; (ns): *p* ≥ 0.05. ctrl: control; SP: SPIONs; unstim: unstimulated T cells; stim: stimulated T cells.

The ability of loaded T cells to perform an immune reaction after stimulation is mandatory. We polyclonally stimulated SPION-loaded and non-loaded T cells with CD3/CD28/CD2 T-cell activator mix overnight. Subsequently, cells were intracellularly stained for cytokine expression such as INFγ, TNFα, and IL-2. We found that neither the loading with SPIONs induced cytokine production nor inhibited cytokine expression for both CD4+ and CD8+ cells after polyclonal stimulation (Figure 6).

## 4. Discussion

The loading of cells with SPIONs enables their control by external magnetic forces. This strategy has been applied for a long time for magnetic associated cell sorting (MACS) in vitro [40] but also represents a great potential for in vivo applications, e.g., for targeted adoptive T cell therapies. For MACS, cells are usually labeled via antibody-conjugated magnetic microbeads, thus, cell labeling enables the isolation of wanted (touched) or unwanted (untouched) cell populations. In contrast to direct targeting of surface markers by antibody-labeled nanoparticles, we use nanoparticles with a citrate shell without any specific targeting moiety. With this, cells can be labeled independently from specific surface structures. Our aim is to make T cells magnetically attractable to enable site-directed targeting for biomedical applications. For this purpose, T cells must be loaded with sufficient iron and at the same time, the cellular function must not be impaired. Here we analyzed how experimental conditions influence nanoparticle uptake and cell viability.

Our SPIONs have a negative zeta potential of around −50 mV at pH 7.3 and a hydrodynamic diameter of 54 nm in water (Table 1). When exposed to physiological fluids, it is known that the nanoparticle surface is immediately covered by various biomolecules to lower the surface energy [41,42,43]. Rocker et al. showed that serum proteins such as the abundant albumin, bind nanoparticles [44]. This protein adsorption, referred to as protein corona, essentially determines the “biological identity” of the nanoparticle, which can also explain reduced targeting efficacy of, e.g., antibody-conjugated nanoparticles in vivo [45]. With 10% FCS in the cell culture medium, our SPIONs remained colloidally stable and roughly 1.4 pg iron was associated with the cells after 3 h of incubation (Figure 1 and Figure 2). Reduction of the FCS content in cell culture medium to 2% led to nanoparticle agglomerations and their sedimentation (Figure 1). In parallel, we observed increased cellular levels of iron (25 pg/cell), which was probably caused by insufficient removability by washing due to nanoparticle sedimentation. In PBS with 2% FCS, in fact, no nanoparticle agglomerations were detected, but cellular iron amounts also increased to nearly 3 pg/cell. Thus, in conditions with 2% FCS only, iron uptake was enhanced but also accompanied by a strong reduction in cell counts (Figure 2). These findings are in line with previous reports by Lesniak et al., who showed much more nanoparticle uptake in the absence of proteins. Analyzing the composition of the protein corona, they found that in the absence of proteins nanoparticles tend to cover themselves with proteins from the plasma membranes, which was exhausting for the cells [46]. This, and the tendency of SPION-loaded cells to aggregate, might have led to a loss of cells in conditions with low protein amounts [47]. Zupke et al. also reported that the presence of human serum proteins reduces the uptake of nanoparticles by CD4+ T cells but preserves cell viability [36].

Besides protein amount, the activation status of the cell was reported to also influence nanoparticle uptake. Others have shown increased incorporation of nanoparticles by activated lymphocytes compared to freshly isolated ones [31,36]. This has been ascribed to the increased macropinocytosis by primary mouse and human T cells after polyclonal activation to support T cell growth [48]. Based on these findings, we polyclonally stimulated T cells to foster nanoparticle uptake. As expected, the T cell count was increased after 72 h, but the stimulated cells did not contain more nanoparticles than the resting ones (Figure 3). Contrary to our data, polyclonal activation of the T cells by anti-CD3, anti-CD28, IL-2, and concanavalin A has previously been shown to increase nanoparticle uptake [49]. In this context, the dependence of nanoparticle uptake on the cell cycle has been discussed. Although cells in different phases of the cell cycle internalized nanoparticles at similar rates, cells in the G2/M phase contained more nanoparticles than those in the S or G0/G1 phase. As soon as cells split, cell-associated nanoparticles were divided between daughter cells [39] and, therefore, it was discussed that increased amounts of nanoparticles were rather due to cell size and the number of endosomes than the cell cycle state [50,51].

When we stimulated T cells after SPION uptake, we found that the release of the cytokines IFNγ, TNFα, and IL-2 was not influenced (Figure 6). Thus, these data confirm earlier investigations of our group, that activation after polyclonal stimulation of human primary T cells was not impaired by SPION loading [27]. In addition, others have previously shown the biocompatibility of SPIONs [30,31,32,36].

To analyze the subcellular location of nanoparticles after incubation with T cells in a cell culture medium containing 10% FCS, we performed TEM. Although it has been described that the internalization of cationic nanoparticles is more efficient in comparison to neutral or anionic nanoparticles due to the interaction with the positively charged plasma membrane [52,53], we found not only a strong association with the plasma membrane but also remarkable amounts of particles intracellularly enriched in vesicles (Figure 4A). The nanoparticles associated with the plasma membranes were rather bound as thin layers, not in clusters. When comparing with the TEM pictures from others using cationic SPIONs, which achieved up to ten-fold higher iron amounts per cell, their nanoparticles were not taken up but attached to the plasma membrane in multiple layers and clusters, with the risk of detachment from the cells [31]. Since lymphoid cells are non-phagocytic, the uptake of nanoparticles has been frequently described to be low and supporting techniques such as functionalization of the nanoparticles by RGD peptides, coatings derived from viruses, or electroporation of the target cells, were employed for better engulfment [54,55]. The difference in the uptake of nanoparticles dependent on incubation temperature, however, indicated an energy-dependent uptake process with the involvement of endocytic processes for T cells as well [36].

When analyzing the SSC as a marker for nanoparticle uptake of co-incubated loaded and non-loaded cells, we found that SSC of the single-cell populations did not change; indicating that SPIONs did not spill from loaded cells to initially non-loaded ones. However, when analyzing the iron amount in the loaded T cells, the AES measurements revealed a reduction in iron during the 72 h observation time, which was not due to cell division, since the analysis was performed with non-proliferating T cells. Others have previously characterized the export of nanoparticles from T cells as an energy-dependent active process, taking part within 24 h, which was reduced by lowering of the temperature or use of inhibitors of cell metabolism [36]. Concerning the cellular persistence of SPIONs, both the retention for several days as well as bisection of the MRI signal within the first 24 h has been reported [56,57,58]. Whereas SPIONs are superparamagnetic, their degradation products, mainly ferritin and hemosiderin, were anti-ferromagnetic, exhibiting a detectable, but reduced MRI signal [59]. Others previously analyzing the degradation of citrate-coated SPIONs in stem cell spheroids found endosomal degradation and upregulation iron homeostasis genes coding for ferritin light chain (iron loading) and ferroportin (iron export) from day 3 onwards [60]. Moreover, after i.v. injection of radioactively labeled SPIONs in mice, after 7 days, 59Fe from the administered nanoparticles appeared in the hemoglobin of newly formed erythrocytes, indicating the intracellular degradation of the nanoparticles. From studies applying SPIONs as contrast agents (Endorem, Resovist), the phenomenon of Fe incorporation into erythrocytes has been well-known [61]. Concerning T cells, it has been found that endosomal acidification was slower and not as robust as in other cells [62]. If SPIONs, in our case, are excreted from the T cells or degraded intracellularly in the lysosomes remains, so far, elusive and must be further investigated. Nonetheless, we detected no spilling to initially non-labeled cells (Figure 4B).

Finally, we analyzed the magnetic retention of the SPION loaded T cells under flow conditions. In magnetic accumulation, the viscosity of the medium, cell radius, and cell velocity are known to play a role [47]. When a permanent magnet generates a magnetic field of about 10–50 T/m over a distance of 1 cm, with a 10 pg iron load, a cell experiences a corresponding force of 1 pN to a few nN [63]. For the investigation of the magnetic accumulation of SPION loaded cells, we used a flow rate of 9.6 mL/min for 1 h at a channel height of 0.4 mm, and a 5 × 5 mm sized permanent neodymium magnet with approximately 400 mT. With this experimental setup, we were at least able to show in vitro magnetic retention of the SPION-loaded T cells in the wanted area (Figure 5). As with magnetic drug targeting, we are aware that magnetic targeting of cells to the tumor region in vivo is much more ambitious and faces several challenges and pitfalls [64,65,66,67]. Exemplarily, tumor vascularization may be unfavorable, hindering the delivery of the cells or nanoparticles for drug delivery. However, others previously functionalized lymphocytes with magnetic nanoparticles and showed increased therapeutic success with the adoptive transfer of T cells and NK cells [28,35]. Further, contactless magnetic movement by permanent magnets and dynamically programmable magnetic fields is under intense investigation [29,68]. Interestingly, for future translation into clinics, the magnetic field coils inherent to clinical MRI scanners can not only be used for tracking but also for steering magnetic nanoparticles or nanoparticle-loaded cells into the wanted region [23,24], possibly enabling an image-based therapy in the future.

## 5. Conclusions

Adoptive T cell transfer suffers from poor efficacy in many patients with solid cancers, which is due to low infiltration of the cells into the tumor. The stable and effective loading of T cells with SPIONs enables their magnetic controllability and accumulation. In summary, we showed here that we can load primary human CD3+ T cells with SPIONs. We found that loading efficacy and cell viability was dependent on the amount of serum present in the cell culture medium. The activation of the cells, however, did not affect nanoparticle uptake. With our loading strategy, we achieved 1.4 pg Fe/iron per cell, which was enough to accumulate the cells in a dynamic flow system. Furthermore, we found that SPIONs were located intracellularly in vesicles or tightly attached to the plasma membrane, without spillover to non-loaded cells. With stable and sufficient iron loading, T cells become not only magnetically controllable but can enable tracking of injected cells using MRI.

## Figures and Tables

**Table 1:** Physicochemical characterization of SPIONs.

Physicochemical Feature	Batch 1	Batch 2	Batch 3
Magnetic susceptibility (10^−3^)	4.08 ± 0.00	4.12 ± 0.00	4.10 ± 0.00
Z-average size (nm) in H_2_O	58 ± 0.1	52 ± 0.1	53 ± 0.2
Polydispersity index (PDI)	0.143 ± 0.005	0.151 ± 0.08	0.152 ± 0.07
Zeta potential (mV) at pH 7.3	−48.5 ± 0.5	−53.7 ± 0.4	−51.8 ± 0.3

## Data Availability

No new data were created or analyzed in this study. Data sharing is not applicable to this article.

## Supplementary Materials

The following are available online at https://www.mdpi.com/article/10.3390/cancers13164143/s1: Supplementary Figure S1: Efficacy of T cell isolation using IBA CD3 Fab-TACS gravity columns.
