# Supplementary figures and images for: Citrate-Coated Superparamagnetic Iron Oxide Nanoparticles Enable a Stable Non-Spilling Loading of T Cells and Their Magnetic Accumulation
Source: Cancers (Basel). 2021 Aug 17;13(16):4143. doi: 10.3390/cancers13164143 (PMC8394404; doi:10.3390/cancers13164143)

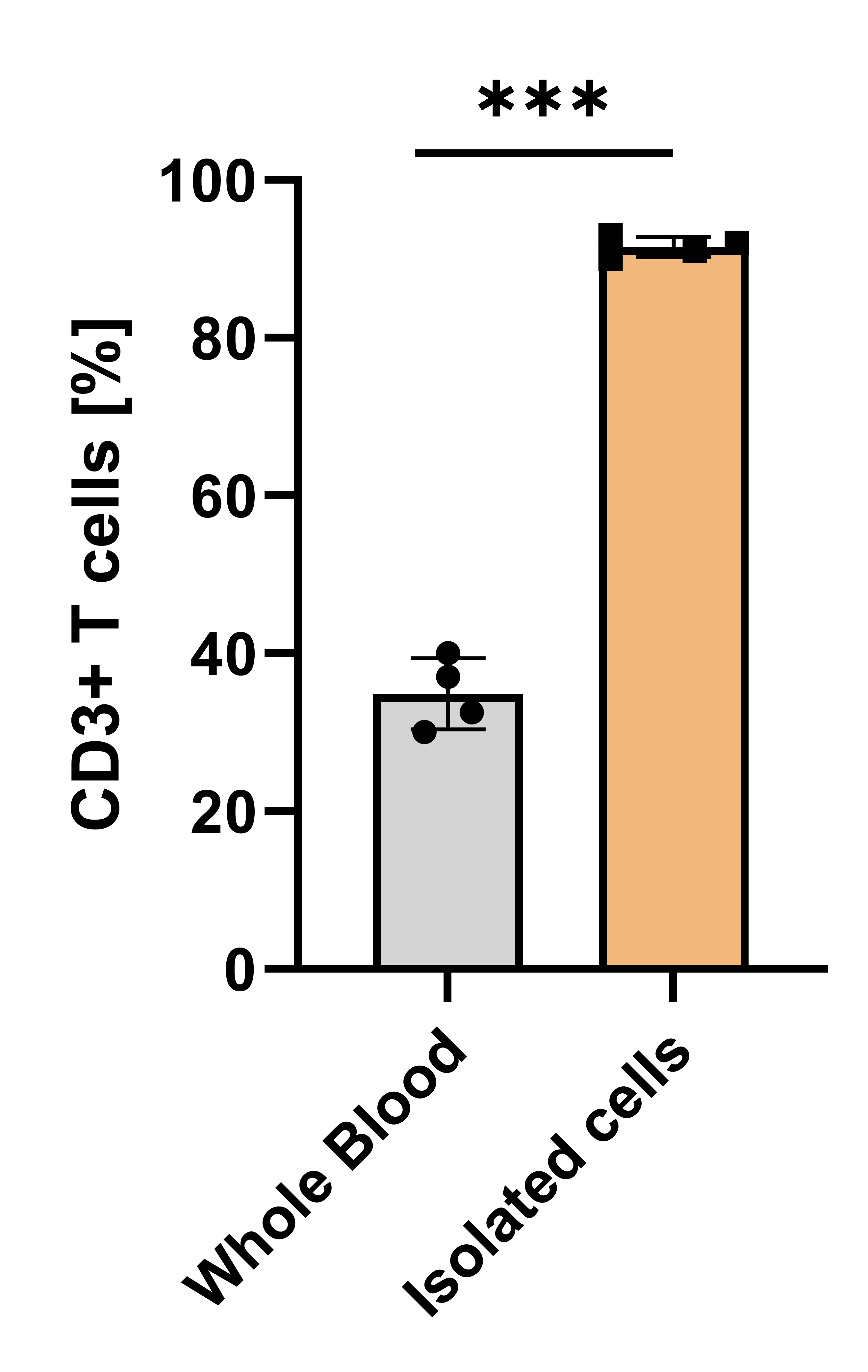

Supplement: Supplementary file 1 [file cancers-13-04143-s001.zip › cancers-1279537.png]
